# Integrating philosophy, policy and practice to create a just and fair health service

**DOI:** 10.1136/medethics-2020-106853

**Published:** 2020-10-07

**Authors:** Zoe Fritz, Caitríona L Cox

**Affiliations:** 1 The Healthcare Improvement Studies Institute (THIS Institute), University of Cambridge, Cambridge, UK; 2 Acute Medicine, Cambridge University Hospitals NHS Foundation Trust, Cambridge, UK

**Keywords:** distributive justice, ethics, public health ethics, political philosophy

## Abstract

To practise ‘fairly and justly’ a clinician must balance the needs of both the many and the few: the individual patient in front of them, and the many unseen patients in the waiting room, and in the county. They must consider the immediate clinical needs of those in the present, and how their actions will impact on *future* patients. The good medical practice guidance ‘Make the care of your patient your first concern’ provides no guidance on how doctors should act when they care for multiple patients with conflicting needs. Moreover, conflicting needs extend far past simply those between different patients. At an organisational level, financial obligations must be balanced with clinical ones; the system must support those who work within it in a variety of roles; and, finally, in order for a healthcare service to be sustainable, the demands of current and future generations must be balanced.

The central problem, we propose, is that there is no shared philosophical framework on which the provision of care or the development of health policy is based, nor is there a practical, fair and transparent process to ensure that the service is equipped to deal justly with new challenges as they emerge. Many philosophers have grappled with constructing a set of principles which would lead to a ‘good’ society which is just to different users; prominent among them is Rawls.

Four important principles can be derived using a Rawlsian approach: equity of access, distributive justice, sustainability and openness. However, Rawls’ approach is sometimes considered too abstract to be applied readily to policymaking; it does not provide clear guidance for how individuals working within existing institutions can enact the principles of justice. We therefore combine the principles derived from Rawls with Scanlonian contractualism: by demanding that decisions are made in a way which cannot be ‘reasonably rejected’ by different stakeholders (including ‘trustees’ for those who cannot represent themselves), we ensure that conflicting needs are considered robustly.

We demonstrate how embedding this framework would ensure just policies and fair practice. We illustrate this by using examples of how it would help prevent injustice among different socioeconomic groups, prevent intergenerational injustice and prevent injustice in a crisis, for example, as we respond to new challenges such as COVID-19.

Attempts to help individual doctors practise fairly and justly throughout their professional lives are best focused at an institutional or systemic level. We propose a practical framework: combining Scanlonian contractualism with a Rawlsian approach. Adopting this framework would equip the workforce and population to contribute to fair policymaking, and would ultimately result in a healthcare system whose practice and policies—at their core—were just.

## Introduction

Healthcare systems face ‘competing, and sometimes conflicting, demands’^1^; resolving conflicts fairly between groups with differing priorities presents a significant challenge. The good medical practice guidance ‘Make the care of your patient your first concern’^2^ provides no guidance on how doctors should act when they care for multiple patients with conflicting needs.^3^ Conflicts are not always a matter of resource allocation—issues surrounding how services are structured, how staff are treated and how information is shared can also be sources of tension.

To address these conflicts, ethical guidance has been drawn up to offer at best help and, at worst, post hoc justification for policymakers. Often ethical goals (eg, treating people fairly) are elided with executive virtues (eg, being flexible). *The central problem, we propose, is that there is no shared philosophical framework on which the provision of care or the development of health policy is based, nor is there a practical, fair and transparent process to ensure that the service is equipped to deal justly with new challenges as they emerge*.

Perhaps because of this, there are many examples where current practice is unjust at local, regional, national and intergenerational levels. Take the recent response to COVID-19, which saw prioritisation of those being treated and working in acute care over those in primary care and care homes. Look at discrepancies in accessing care (and information about care) between those in different socioeconomic groups, even within the same region. Consider the depleted workforce which future patients will face because of poor investment in maintaining our nurses and doctors.

We believe that to achieve justice and fairness in practice and policy, a philosophical framework must be made explicit, in particular to help guide the development of principles which treat groups with different demands fairly. We believe that if we develop and embed such a framework for policy decisions, then the individual decisions which clinicians make on a day-to-day basis will also become fairer and cause less moral discomfort. If the process for considering and weighing conflicting demands is fair, then the resulting outcomes will be just.

Many philosophers have grappled with constructing a generalisable set of principles which would lead to a ‘good’ society which is just to different users, notably John Rawls.^4–6^ While his theory has previously been rejected as being useful in resolving *individual* issues in healthcare,^7^ we have argued that it can provide a useful framework for making policy decisions, guiding how a just health service should be constructed and sustained.^8^ Daniels has also previously drawn on Rawls: he argued, in ‘Just Health’, that resource allocation decisions can be made fair by ensuring that they are public, relevant, amenable to appeals and that the process is regulated.^9^ Badano argued that the ‘relevant’ condition rendered this process too unfair for individuals, whose interests are sacrificed for the sake of groups.^10^ We note it also fails to recognise the importance of intergenerational justice. Like Daniels, we argue that Rawls can be used as an example of procedural justice to make healthcare decision-making more just; we extend Daniels’ argument (which centres on how resources can be allocated) to other aspects of healthcare policy, to fairly balance the needs of a range of users in this, and future, generations.

There is a significant challenge in creating a framework which is philosophically robust, yet easily accessible to politicians and clinicians, and which can be fairly applied to create just healthcare policies. In this essay, we will demonstrate how this can be done by combining Scanlonian contractualism with explicit principles derived from a Rawlsian framework.

First, for those unfamiliar with it, we will summarise Rawls’ Theory of Justice and the principles which emerge from it, and discuss how it could be used as a philosophical framework to guide the development of a just healthcare system. We will then consider practical difficulties with implementing this Rawlsian approach, and examine the benefits (and problems) of using Scanlonian contractualism as an alternative. We will explore how combining a Rawlsian framework with elements of Scanlonian contractualism could helpfully guide decision-making within the National Health Service (NHS) as it stands. Finally, we will illustrate, with examples, how this might be used to approach conflicting demands facing the health service.

## A Rawlsian approach for the health service

John Rawls’ ‘Theory of Justice’ aims to determine a set of principles which, if followed, will give rise to a just basic structure for society.^4^ Rawls imagines people in what he terms the ‘original position’, where they are behind a veil of ignorance. Behind the ‘veil’, people do not know what their position in society will be—they are ignorant as to their race, gender, wealth, natural endowments and religious/political convictions.^4^ Rawls asks what principles ‘*free and rational persons concerned to further their own interests*’ would agree to from this position. People in this conception are ‘*capable of reasonableness*’, and will act on whatever principles are agreed to. The terms that such agents would agree to from behind the veil are, by virtue of their agreement alone, just. Rawls uses the phrase ‘justice as fairness’ to reflect this principle: it ‘*conveys the idea that the principles of justice are agreed to in an initial situation that is fair*’,^4^ https://www.gov.uk/government/groups/moral-and-ethical-advisory-group%23meeting-summaries

Rawls argues that, by using this procedure, two main *principles of justice* would emerge:

1. All persons should have equal basic liberties.
2. Social and economic inequalities (of ‘*primary goods* ^1^’) need to satisfy two conditions:
  - They should be attached to positions/offices open to all under fair equality of opportunity.
  - Primary goods should be distributed equally, unless an unequal distribution would be to everyone’s—and in particular the worst-off’s—advantage (known as the ‘*difference principle*’).

Rawls addresses intergenerational justice by making people in the original position ignorant to the position that their own generation holds in the timeline of generations. Someone behind this version of the veil would not agree to a system in which early generations are able to use resources at an unsustainable rate, to protect themselves from the possibility of being in a much later generation with a paucity of resources. Yet they would also not agree to a system where early generations are forced to be so frugal with resources that they cannot make use of them at all. Rawls terms this balance the ‘*just savings principle*’, which he describes as ‘*an understanding between generations to carry their fair share of the burden of realizing and preserving a just society*’.^4^ The difference principle determines distributive justice *within* one generation, while the just savings principle determines resource distribution *between* generations. The just savings principle thus constrains the difference principle, and emphasises that people have a duty to create a system which permits the realisation and maintenance of a just basic structure over time.^6^

We previously applied this approach to healthcare and asked what a health service would look like if those making policies did not know if they were a patient, healthcare worker or manager—in this generation or the next.^8^ We concluded that its basic structure would bear similarities to the NHS as it currently exists (it would provide comprehensive services which are free at the point of need) but it would not be identical. Two important general principles emerge in addition to equity of access; we believe that articulating and emphasising these in policymaking would lead to a more just health service.

First, in considering examples where the immediate needs of individuals conflict with the future needs, it is clear that there is a strong requirement for a healthcare service which is *sustainable* (in its training and treatment of staff, and in encouraging research). Second, *increased openness* is required: since power and opportunities are primary goods, individuals—both healthcare professionals and the public—should have the opportunity to exert some power over the system which provides their healthcare. The system must thus be open in terms of its *transparency* of decision-making to patients and staff, and *accountable* in terms of the individuals and organisations which make decisions about the running of the health service.

**Figure 1:**
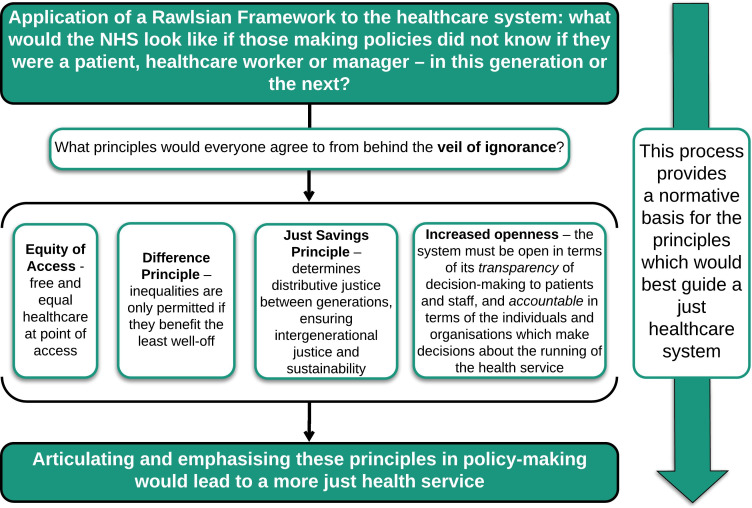
Applying a Rawlsian framework to the healthcare system. NHS, National Health Service.

The process of applying a Rawlsian analysis to the health service could provide a normative basis for the principles that would best guide the development of a just health service. As demonstrated in figure 1, four important principles can be derived: equity of access, the difference principle, the just savings principle and openness.

It is important to consider to whom these principles apply, something which Rawls termed the ‘subject’ of justice.^4^ For Rawls, the primary subject of justice is the *basic structure of society—*the major political, social and economic institutions, which act as public regulatory structures. Rawls focuses on the justness or unjustness of institutions or social systems, rather than the actions of individuals. Indeed, Rawls ‘*does not consider justice as a personal virtue, but the first virtue of social institutions*’^11^; the principles of justice should not be applied to personal conduct, but to the rules which constitute the basic institutions. We thus argue that the principles to guide a just healthcare system (as outlined in figure 1) do not apply to the individual actions of those working within or using the NHS, but to the structures which make up the health service itself. As such, the principles do not necessarily provide specific guidance on how individual issues should be managed; they provide broader guidance on how a just health service should be constructed and maintained.

The focus of Rawls’ Theory of Justice on institutions, as opposed to individuals, can make it challenging to implement in practice. As Shevory has noted, Rawls’ work ‘*is highly abstract and leaves to others the task of matching real-world circumstances to ethical principles consistent with the overall theory*’.^7^ Rawls relies on an abstracted veil of ignorance to develop principles which should be applied to the institutions in order to produce a just system in ideal circumstances. It is not always clear how actual agents, who exist in non-ideal conditions, should use his principles to guide the regulation of existing institutions.

Perhaps the most pressing question for us, then, is how a Rawlsian approach can be applied in practice to guide policy and resolve conflicts within the existing NHS. Individuals who make healthcare policy decisions need the above principles, and guidance on how to practically apply them to existing institutions to make the system more just. To do this, we combine Rawls with elements of Scanlonian contractualism.

## Scanlonian contractualism

Scanlon argued that just principles could be derived from debate between individuals who are motivated by a desire for reasonable agreement, acting in a way which others could not ‘reasonably reject’^12^ (in contrast with Rawls’ ideal of finding principles which everyone would agree to). The agents in Scanlon’s model are aware of their self-interests and position in society. They wish to justify themselves to others (who have their own interests to pursue); if an action can be reasonably rejected by those affected by it, by Scanlon’s definition, it is wrong. This theory thus appeals to the normative notion of reasonableness to produce a theory of *interpersonal obligations*, focusing on what we owe to each other.

Why, then, should we not simply rely on Scanlonian contractualism as a way of developing just principles for a healthcare service? We argue that there are several key advantages to applying a Rawlsian approach to consensus building and reasonableness.

One issue with Scanlon’s approach is that any individual group can only represent their current interests—it does not lend itself as well to intergenerational conflicts, as only the views of current persons are considered. Some have argued that Scanlonian contractualism *can* be extended to consider the rights of future persons, as ‘*the fact that future people do not co-exist with us…does not prevent us from hypothetically contracting with them by considering them among those to whom we have reason to justify principles*’.^13^ We could thus ask whether future people could reasonably reject the principle when considering the morality of an action. Overall, however, Rawlsian justice has been applied to intergenerational conflict more widely, and more compellingly, than Scanlonian contractualism has.^14 15^ Problems where the rights of current users must be balanced with future users of a healthcare system are more convincingly addressed using a Rawlsian approach, which explicitly considers the issue of sustainability through discussions of how just institutions should be maintained across time. Applying Rawls’ just savings principle would, for example, provide justification for research and innovation in the NHS to improve the service provided to future generations, even if there is some cost to current generations.

A further problem is that Scanlonian contractualism aims to produce a general moral theory to determine what we owe to each other, but does not defend any substantive principles of distributive justice. Scanlon aims to develop ‘principles of right and wrong for *individual* actions in such a way that the interests of each affected person are taken fairly into account’,^16^ while Rawls focuses on the basic institutions and aims to develop principles which, if followed, would result in a just society. In this sense, Scanlonian contractualism might be helpful in guiding reasoning about specific individual healthcare decisions (and has already been suggested to guide decision-making around; eg, broad-spectrum vs narrow-spectrum antibiotic use^17^), but is less useful than a Rawlsian approach in guiding the overall development of a just healthcare system. An example of this can be seen in the application of Rawls’ work to justify publicly funded universal access to healthcare, most notably by Daniels.^9^ Rawls’ Theory of Justice applies to the institutions which make up the basic structure of society; in this way, it can be of help in justifying universal access to healthcare in a way that Scanlon’s work, focused as it is on interactions between individuals, cannot.

## A practical solution: combining Scanlonian contractualism with a Rawlsian approach

We have shown that relying purely on the work of either Rawls or Scanlon alone would not provide a practically useful framework to guide policy. Rawls’ Theory of Justice relies significantly on the hypothetical veil of ignorance; the principles derived from it could be applied to the institutions making up a healthcare system, but can be difficult for policymakers working within existing institutions to enact. Scanlon’s concept of self-interested contemporaneous agents debating principles is more practically applicable as it does not demand the veil of ignorance, but it is overly focused on what individuals owe each other. Consequently, it does not provide as compelling an account of how to create a healthcare system which is just to current and future generations.

**Figure 2:**
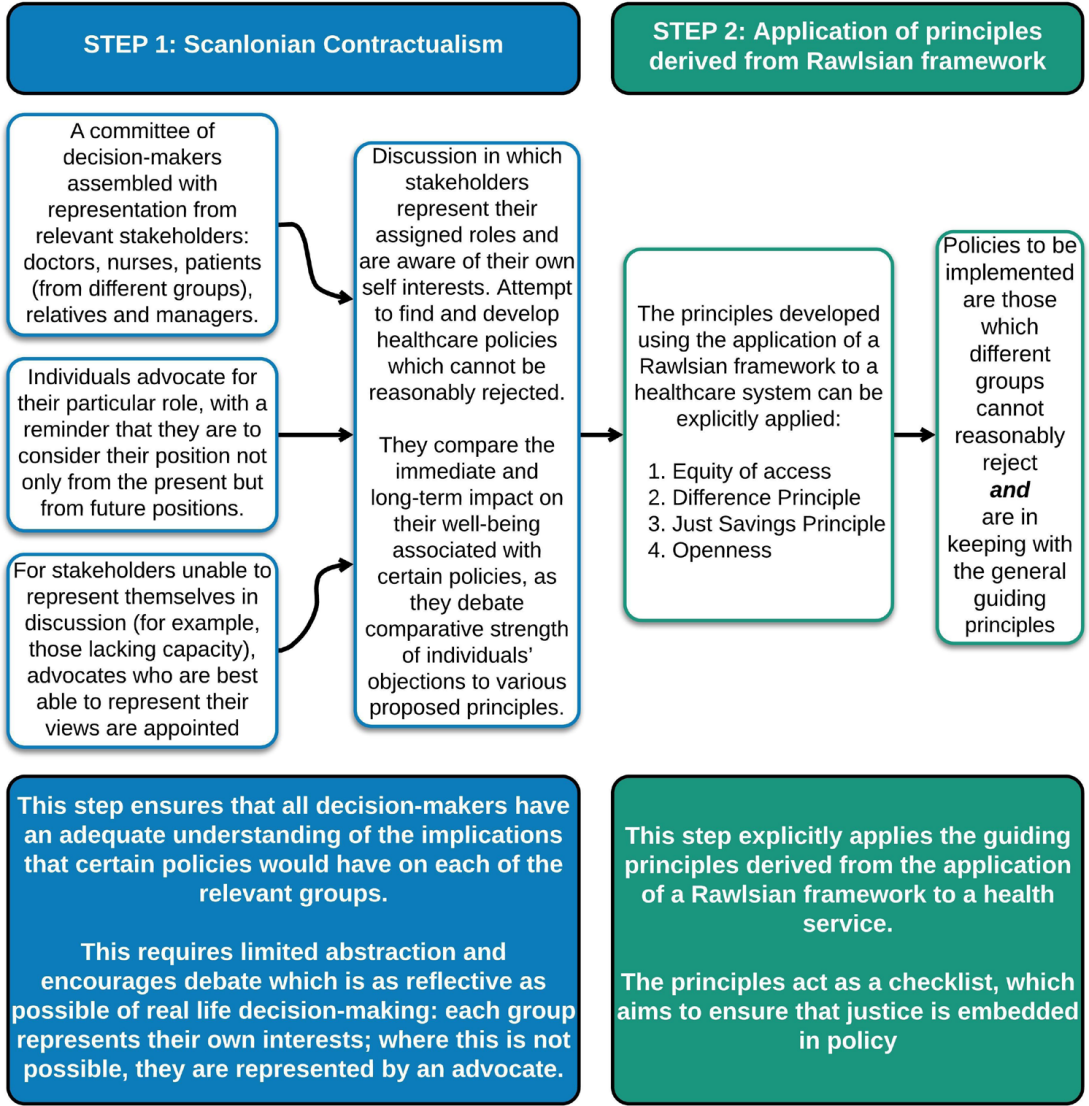
A two-step process to provide a practical approach to healthcare policymaking.

We thus propose a method of implementing an adapted Rawlsian approach for the NHS by combining it with some elements of Scanlonian contractualism, as demonstrated in figure 2. Scanlonian contractualism could initially be used: a committee of healthcare workers, patients, relatives and managers could come together as themselves and represent their own interests, contemporaneously discussing how different policies might impact on them (with a reminder to consider from present and future positions). To ensure that those less able to represent themselves (or at all) were represented, additional independent advocates would be appointed to participate in the process. Scanlon refers to this as the ‘trustee model’, in which objections to certain principles could be raised by ‘trustees’ representing those who themselves lack the capacity to assess reasons and express objections (eg, infants or the cognitively impaired).^12^

In this way, Scanlonian contractualism would elucidate the impact of actions on different stakeholders, as assessing ‘the comparative strength of individuals’ objections to various proposed principles will centrally involve comparing the immediate and long-term gains and losses to their well-being’.^18^

The second step entails the committee of stakeholders explicitly considering if policies being considered are in keeping with the general principles derived from the Rawlsian approach. The principles themselves come from applying the ‘veil of ignorance’ to the healthcare service’s institutions as a whole (see figure 1); here they act as a checklist to be considered by the stakeholders when making decisions or drawing up policies. This step ensures that each policy decision is examined to see if it meets the principles of distributive justice, openness and sustainability.

We recognise, of course, that there are already many examples of engaging with patients and of ‘stakeholder involvement’. Several of these have gone some way towards making healthcare practice and policy more just: patient and public involvement in research planning and execution; lay membership on boards; including multiple stakeholder perspectives in decision-making around healthcare rationing^19^ and ongoing efforts to ensure that individuals with a range of ethnicities and genders are included in many committees and groups. Citizens’ assemblies, such as those held in Ireland, have been convened to consider important policy decisions, but this has not frequently happened in healthcare.

While these efforts to widen representation in the decision-making body are laudable, it is not the same as explicitly requesting that different groups they have their interests considered in a Scanlonian way. Efforts to include different stakeholders in decision-making do not generally encompass the ‘reasonable rejection’ criteria, which we have proposed in the first step of our model. By demanding that decisions are made in a way which cannot be reasonably rejected by stakeholders, we ensure that their needs are considered far more robustly.

How then might this approach be embedded to help ensure just policies and fair practice, and how might it be applied to help the NHS deal flexibly with new challenges such as COVID-19?

## Applications of the framework

### Preventing injustice among different socioeconomic groups

There are many recognised injustices in access to and delivery of healthcare across socioeconomic groups. We recognise that this inequity is acknowledged and periodically investigated, and that it is difficult to meaningfully measure whether interventions to change socioeconomic disparities have direct effects on health outcomes.^20^ However, our proposal will provide a stop check to consider whether new policies (unintentionally) worsen these inequities. The process of explicitly ensuring advocacy for those who may be less able to articulate or argue their own case will help make policies fairer for them (see figure 2); the check to ensure equity of access will help keep this at the forefront as policies are made. We recognise that, to some extent, this is already done: efforts are made to seek representation when new health services are being developed to ensure that they are accessible to those who need them most. But there is a distinction between consulting with a representative and ensuring that their view cannot be reasonably rejected. We argue that there is a value in explicitly applying the ‘difference principle’ so that if a change (eg, the location of a new health provider, or a new screening programme) disproportionately benefits one group in society, it benefits that group which is currently most disadvantaged.

### Preventing intergenerational injustice

Currently policies are often made without explicit consideration of the impact on future generations. National policies are driven by a ‘quick return’ in reaction to the most pressing problem, and to ensure that voters will see the results of the actions taken and re-elect those in power. There is no obligation or accountability to consider the impact of policy decisions on future generations, and little incentive to do so. The strength of explicitly acknowledging Rawls’ ‘just saving principle’ as part of our proposed framework (see figure 2), means that sustainability is considered more robustly. The 2015 policy of removing student grants for nurses, for example, led to a 30% drop in applications: in 2019 the nursing shortage in England had increased to 40 000.^21^ This policy, which led to immediate savings but could be confidently predicted to result in a depleted nursing workforce, is clearly not in keeping with the just savings principle.

In addition to preventing short-sighted policies, our framework would give voice and power to policies which involved proactive planning for a fairer future: recruiting staff to specialities or regions which were underserved, or investing in preventative medicine and research to prepare for future health crises. The model we propose here represents the inclusion of a step to ensure that policies are designed to establish and preserve just healthcare institutions over time.

### Preventing injustice in a crisis

While our proposed framework can be applied to deal with challenges which we are already aware of, it becomes even more useful when an unexpected challenge arises. Instead of having to start from first principles, an established method of reasoning and a set of accepted principles can be drawn on. This would have been invaluable in approaching the COVID-19 pandemic.

Radical changes had to be made to the health service in light of COVID-19, and many of the decisions necessitated balancing conflicting needs from different users. We suggest that a fundamental problem in the response to the crisis was the lack of clear philosophical framework or just decision-making process. Of note, the UK government’s Moral and Ethical Advice Group met, but only very brief notes were published: in them, there was no reference to a process by which decisions were considered, nor of the use of philosophical principles.^22^ This lack of coherent ethical framework guiding policy decisions was evident in the—at times—narrowly focused and frantic attempts to restructure services.

At the beginning of the pandemic in the UK there was a fear—based on experiences in Italy—that there would be an insufficient number of ventilators. Significant attention was thus bestowed on the demands of the most critically ill: the nightingale hospitals were built at pace, and multiple ventilator projects were spawned. Those in care homes and in primary care were, certainly in the early stages of the response, overlooked. Vulnerable patients were discharged back to nursing homes where they may have spread disease, while primary care and care home staff were left with insufficient personal protective equipment.

Had our framework been used, nursing home residents and staff would have reasonably rejected these policies. The needs of patients without COVID-19 might also have been recognised earlier had their voice (or that of their ‘trustee’) been included in debates. Suspending cancer screening services or cancelling urgent surgeries might have been reasonably rejected by those who needed this care. The requirement for sustainability would also have encouraged policymakers to think about developing alternative streams for these patients, away from COVID-19 admission units, rather than putting off the problem for a future date. Finally, those working within the health service were often given little opportunity to challenge policies or contribute to decision-making: the principle of openness was not adhered to, and the resulting decisions were weaker as a result.

A full exploration of exactly what policies might have been adopted using our suggested framework is beyond the scope of this essay; we would instead like to highlight that the way some decisions were made did not afford adequate consideration to how they would impact a range of health service users and workers—both present and future—including those without COVID-19 and those in primary care or care homes. Had the proposed framework been in place, then the impact of policies on all stakeholders would have been fairly considered, including minority groups and the most vulnerable in society. An explicit consideration of the principles of distributive justice, openness and sustainability could have provided an ethical basis for guiding policy made in response to the challenges of COVID-19.

## Conclusion

We propose that attempts to help individual doctors practise fairly and justly throughout their professional lives are best focused at an institutional or systemic level. At the moment, individual clinicians face moral discomfort as they make decisions within a healthcare system which is at times unfair: they refer a patient to their local hospital knowing it is less well resourced than the one 100 miles away; they work with colleagues whose training has been compromised and whose pay is inadequate; they discharge patients who are potentially transmitting infectious diseases back to care homes to make way for more acutely sick patients.

Establishing guidance that will help doctors negotiate specific cases of injustice is challenging: every situation is different. Moreover, while individual policies to address each injustice could be constructed, this would not solve the problem: more unexpected injustices would appear, as we have unfortunately just witnessed.

We recognise that implementing a philosophical framework to guide just healthcare policy would not suddenly remove all unfairness in the system, and doctors would still sometimes find themselves in situations where their actions might seem unfair to current or future users of the health service. Yet by introducing a just process for policymaking, clinicians would less frequently find themselves in such situations. If clinicians were confident that the policies being made were based on a consistent and robust ethical framework—and one which was open to engagement and challenge—then their individual decisions would become less morally challenging.

At the moment, there is a striking absence of an underlying philosophical framework on which healthcare policy decisions within the NHS are made. This has resulted in a system which is not always just in its resolution of conflicting demands from different users, current or future. We have proposed a practical framework—a transparent Scanlonian process and a set of principles derived from Rawls (as depicted in figures 1 and 2)—on which the development of health policy could be based, and which could be drawn on as new challenges arise. Having a clear process for the development of healthcare policy, based on philosophical principles and designed to be used in practice, could help make the NHS fairer to all of its users. Adopting this framework would equip the workforce and population to contribute to fair policymaking, and would ultimately result in a healthcare system whose practice and policies—at their core—were just.

## Footnotes

Rawls says, ‘Primary social goods are things which it is suppose a rational man wants whatever else he wants… The primary social goods, to give them in broad categories, are rights, liberties, and opportunities, and income and wealth. ([and]A very important primary good is a sense of one’s own worth)’ (p 79, The Theory of Justice).
